# Physicochemical, nutritional, functional, rheological, and microbiological properties of sorghum flour fermented with baobab fruit pulp flour as starter

**DOI:** 10.1002/fsn3.913

**Published:** 2019-01-29

**Authors:** Abubaker B. Makawi, Abdelmoniem I. Mustafa, Oladipupo Q. Adiamo, Isam A. Mohamed Ahmed

**Affiliations:** ^1^ Department of Grain Technology National Food Research Center Ministry of Science and Technology Shambat Sudan; ^2^ Department of Food Science and Technology Faculty of Agriculture University of Khartoum Shambat Sudan; ^3^ Department of Food Science and Nutrition College of Food and Agricultural Sciences King Saud University Riyadh Kingdom of Saudi Arabia

**Keywords:** antinutritional factors, Baobab fruit pulp, fermentation, sorghum flour

## Abstract

Two sorghum genotypes termed Wad‐Ahmed (high tannin) and Tabat (low tannin) in Sudan were fermented with different starter levels (0%, 25%, 50%, 75%, and 100%) of fermented baobab fruit pulp flour (FBFPF). Chemical composition, antinutrients, extracted minerals, and the microbiological, physicochemical, functional, rheological, and pasting properties from the fermented flours were determined. Fermentation of both genotypes with higher levels of FBFPF starter enhanced protein, fiber, ash, and major mineral contents and extractability (*p *≤* *0.05). Total acidity, bulk density, rheological properties, in vitro protein, and starch digestibility of both genotypes increased with FBFPF levels, with a concomitant decrease in pH, phytate and tannin contents, and water and fat absorption capacities (*p *≤* *0.05). Microbial loads, especially lactic acid bacteria, increased with increasing FBFPF starter levels in both genotypes. Use of FBFPF as a starter in the fermentation of sorghum flour can improve the nutritional value of sorghum. This could be usefully applied to the food industry for the development of fermented sorghum products.

## 1 INTRODUCTION

The recent increase in sorghum production could be due to several factors, such as genetic improvement and introduction of new varieties that are highly productive. Most of the production in Africa and Asia is mainly for human consumption as it is a main source of nutrients for many people (Godwin & Garry, 2000). However, like other cereals, the nutritive value of sorghum is inadequate due to its deficiency in essential amino acids and also the presence of some antinutritional factors, such as tannin and phytate that interact with proteins, vitamins, and minerals and reduce the bioavailability of these nutrients for absorption in the human digestive tract (Ahmed, Mahgoub, & Babiker, 1996). In the gastrointestinal tract, dietary minerals are chelated by these antinutritional factors, reducing their bioavailability and bio‐accessibility (Idris, AbdelRahman, El Maki, Babiker, & El Tinay, 2005).

In African countries, the baobab tree (*Adansonia digitata* L.) has been extensively used for centuries as a source of food, medicine, and fodder (Sidibe & Williams, 2002). In Sudan, the dry pulp of the fruit is consumed fresh or is dissolved in water as a fermenting agent in local brewing or as a sauce in food (Abdalla, Mohammed, & Mudawi, 2010). The fruit pulp is regarded as a good source of vitamin C, pectin, polyphenols (epicatechin and procyanidin), carotenoids, fatty acids (linoleic and oleic), and major minerals including calcium, magnesium, and phosphorus, good quality protein (Kabore et al., 2011). In addition, it has antioxidant, antimicrobial, antiviral, and anti‐inflammatory potentials and has been used extensively in African and Asian traditional medicine to cure several diseases such as diarrhea, microbial infections, malaria, and fever (De Caluwé, Halamová, & Van Damme, 2010). Domestic processing conditions of baobab fruits to produce juice or fermented products have been previously investigated (Parkouda, Ba/Hama, Ouattara/Songre, Tano‐Debrah, & Diawara, 2015; Parkouda et al., 2010; Tembo, Holmes, & Marshall, 2017). Uncontrolled heat treatments and storage conditions have great influences on the bioactive compounds of baobab fruit juice (Tembo et al., 2017). In the natural fermentation of Maari, a fermented baobab seed product largely prepared and consumed in western Africa, several aerobic mesophilic and lactic acid bacteria (LAB) were involved (Parkouda et al., 2010), and the process resulted in several biochemical changes in amino acids and fatty acids of Maari (Parkouda et al., 2015). However, there is insufficient information on the utilization of fermented baobab fruit pulp as starter culture for fermentation of cereal‐based products.

It is well known that exogenous and endogenous enzymes produced during domestic processing can substantially reduce the levels of antinutritional factors, such as phytate and tannin, in the products of some tree fruit, cereals, and legumes. Processing methods, such as dehulling, malting, fermentation, and cooking, can reduce antinutritional factors and improve the nutritional content of cereals and legumes (Idris et al., 2005; Nnam & Obiakor, 2003). In Sudan, the whole baobab seeds are coarsely powdered, fermented, and added to a traditional dish known as Kurundu that consumed with sorghum‐based traditional foods such as *Kisra* and *Assida* (Dirar, 1993). However, there is no report on the impact of fermented baobab seeds on the quality of this traditional food. It is conceivable that fermentation with baobab starter culture is useful in improving the nutritional, antinutritional contents, and functional properties of low and high tannin sorghum genotypes. To address this, the present study assessed the effect of different levels of fermented baobab fruit pulp flour (FBFPF) as a starter on the chemical composition, antinutritional factors, mineral contents and extractability, microbiological characteristics, and the physicochemical and functional properties of high and low tannin sorghum genotypes.

## 2 MATERIALS AND METHODS

### 2.1 Sample collection

Two sorghum (*Sorghum biocolor*) genotypes, locally known as Wad‐ Ahmed (high tannin) and Tabat (low tannin), were collected from the Sinnar Research Station, Agricultural Research Corporation, Sinnar, Sudan. Baobab fruit was obtained from a local market in Khartoum, Sudan. The grains and fruits were carefully cleaned to remove all foreign materials. The grains were then decorticated with an extraction rate of 90%. Seeds of dried baobab fruit were detached from its pulp by using a special high rate mechanical extracting machine. Baobab fruit pulp flour was prepared by grinding the dried pulp to pass through a 0.4‐mm mesh screen and kept at 4°C until used. All reagents and media were of standard analytical grade.

### 2.2 Fermentation process

#### 2.2.1 Fermented baobab fruit pulp flour

Natural fermentation of baobab fruit pulp flour by microflora present in the grain was carried out by mixing the flour with water (1:2 w/v). The mixture was incubated at 37°C for 24 h in a sterile covered flask and then stored at 4°C in tightly closed containers until used for the fermentation of sorghum flour.

#### 2.2.2 Fermentation of sorghum flour with FBFPF as starter

The method of El Tinay, El Mehdi, and El Soubki (1985) was employed in the traditional fermentation (lactic acid fermentation) of the sorghum flour of both genotypes (low and high tannin) as practiced in most Sudanese households. Initially, the sorghum flour was naturally fermented by the original microorganisms present in the grain. This involved addition of flour to water at a ratio of 2:1 (flour: water, 40 g flour:80 ml water), and the dough was kept in the incubator at 37°C for 24 h. The sorghum starter obtained was kept in the refrigerator until used for fermentation of the flour samples. Sorghum flour (180 g) was mixed with 360 ml of water, and then, 60 ml starter obtained from previously fermented dough was added to 540 ml of the flour paste and mixed thoroughly. This was followed by addition of FBFPF starter at different levels (0%, 25%, 50%, 75%, and 100% of FBFPF/60 ml of sorghum starter) to 540 ml dough, and the volume of the sorghum starter was decreased accordingly. The mixture was incubated at 37°C for 24 h in 1‐L sterile conical flask. This was followed by freeze‐drying of the fermented samples (FBFPF, naturally fermented sorghums, and sorghum flours fermented with 25%, 50%, 75%, and 100% FBFPF) for rheological analysis or drying in a hot air oven (Heraeus UT 5042, Germany) at 65°C for 16 h. The samples were then milled to pass through a 0.4‐mm mesh screen and stored at 4°C in tightly closed containers until analyzed. The control samples were Tabat and Wad‐Ahmed sorghum flours (TSF and WSF) fermented with only sorghum starter (60 ml) without addition of FBFPF starter and unfermented (or raw) TSF and WSF.

### 2.3 Chemical composition

The chemical composition of the raw and fermented samples was determined as described in the AOAC (2000) method.

### 2.4 Mineral contents

Mineral contents were determined according to Pearson (1970) with slight modification. Exactly 2 g of sample was heated at 550°C for 3 h and then cooled. The ash obtained was treated with 10 ml of concentrated hydrochloric acid (50% HCl; LabChem, USA) with the addition of 5 ml of nitric acid (33%) and then placed in a water bath (100°C) for 1 h. Ten milliliters of HCl was added, and the sample was returned to the water bath for 15 min. The mixture was then transferred to a 100‐ml volumetric flask containing distilled water with a final volume of 100 ml and well shaken. After sample preparation, mineral concentration was determined. Sodium (Na) and potassium (K) were determined by flame photometer (Jenway PFP 7, Essex, UK) as described by Overman and Davis (1947). Ca, Mg, and iron (Fe) were determined by atomic absorption spectrophotometry (Perkin–Elmer 2380, Norwalk, CT, USA) following the methods described by Cabrera, Lorenzo, and Lopez (1995). Phosphorus (P) was determined by vanadate–molybdate method as described by Chapman and Pratt (1982). Briefly, 5 ml of the mineral extract prepared above was mixed with 10 ml of ammonium molybdate–vanadate reagent and the mixture was kept for 30 min at room temperature. After that, the developed color was recorded by ultraviolet–visible (UV‐Vis) spectrophotometry (Jenway 6305, Essex, UK) at a wavelength of 440 nm and the P concentration was determined from the standard curve generated using different concentrations of P.

### 2.5 Mineral extractability

The HCl extraction of minerals of samples was performed as described by Chauhan and Mahjan (1988). Briefly, 1 g sample was added to 10 ml of 0.03 M HCl and shaken for 3 h at 37°C. The mixture was then filtered, and the clear extract obtained was oven‐dried at 100°C and acid‐digested. The content of the extracted minerals was analyzed as described above. The percentage of HCl extractability was calculated using the formula:

$$\text{Mineral extractability}\;\left(\%\right)=\frac{\text{Mineral extractable in 0.03 M HCl (mg/100 g})}{\text{Total minerals (mg/100 g})}\times 100$$

### 2.6 pH and total acidity (TA) determination

The pH of a mixture of the flour/dough samples and water (2% w/v) was measured using a pH 211 microprocessor pH meter (Hanna, Woonsocket, RI, USA). The TA was determined according to the AOAC (2000) method using a mixture of 2 g of the samples and 10 ml distilled water.

### 2.7 Microbiological evaluation

The total count of bacteria (TCB), LAB, and yeast and mold (YM) counts of the raw and fermented samples was determined as described by Harrigan and MacCance (1976) with slight modification. Briefly, 1 g sample was mixed with 9 ml of sterile peptone water (0.1%). A volume of 1 ml of the homogenate was diluted with 9.0 ml of 0.1% peptone water to prepare serial 10‐fold dilutions. TCB was determined by plating (pour plate method) appropriate serial dilutions in duplicate with plate count agar and incubating at 37°C for 48 h. LAB were enumerated by plating appropriate serial dilutions anaerobically with MRS agar and incubating at 37°C for 4 days. YM counts were done by plating appropriate serial dilutions with malt extract agar and incubating for 48 h at 28°C.

### 2.8 Determination of functional properties

The method of Okezie and Bello (1988) was employed to determine bulk density (BD). Briefly, 10 g sample was placed in a 25‐ml graduated cylinder and packed by gently tapping the cylinder on bench top (10 times) from a reasonable height (5–8 cm). The final volume was recorded, and the BD was expressed as g sample per ml volume occupied. Water absorption capacity (WAC) and fat absorption capacity (FAC) were determined following the method described by Lin, Humbert, and Sosulski (1974) with slight modification. Briefly, 3 g of the sample was added to 30 ml of water for WAC determination while 4 g sample was added to 20 ml of refined corn oil (specific gravity 1.52) for FAC determination in 50‐ml centrifuge tube. The mixture was stirred for 5 min, and the contents were allowed to equilibrate for 25 min. The suspension was then centrifuged at 4,400 *g* for 20 min using a model 5430 apparatus (Eppendorf, Hamburg, Germany). The freed water and oil were carefully decanted, and the volumes were measured. The WAC and FAC were expressed as ml of water and fat absorbed per gram of sample.

### 2.9 Antinutritional factors, in vitro protein digestibility (IVPD), and in vitro starch digestibility (IVSD)

The determination of phytic acid of the samples was carried out as described by Wheeler and Ferrel (1971). Briefly, 2 g sample was extracted with 50 ml of 3% trichloroacetic acid (TCA) for 3 h with mechanical shaking. The mixture was centrifuged, and 10 ml aliquot of supernatant was added to 4 ml of FeCl_3_ solution (containing 2 mg Fe^+3^ per ml 3% TCA) and heated in boiling water for 45 min. Different Fe(NO_3_)_3_ concentrations were used as standard and phytate phosphorous was calculated from the standard curve and was expressed as Fe(NO_3_)_3_ equivalents, by assuming and Fe:P molar ratio of 4:6. The vanillin‐HCl method (Price, Van Scoyoc, & Butler, 1978) was used to determine the content of tannins. Briefly, extraction was carried out by adding 0.2 g sample to 10 ml of 1% HCl in methanol (v/v). The mixture was shaken for 20 min and centrifuged at 2,500 *g* for 5 min. Exactly 1 ml of supernatant was added to 5 ml of vanillin‐HCl reagent (equal volumes of 8% HCl in methanol and 4% vanillin in methanol). A standard catechin solution was prepared and used as reference standard. The absorbance of sample and standard solutions was read using spectrophotometer (Jenway V6300) at 500 nm after 20 min incubation at 30°C. Tannin concentration was expressed as catechin equivalent percentage.

The method of Monjula and John (1991) was used to determine the IVPD of the samples. Briefly, a known sample weight containing 16 mg nitrogen was digested with 1 mg pepsin (activity: 1:3000, Carolina) in 0.1 M HCl (15 ml) for 2 h at 37°C. Fifteen milliliters of 10% TCA was added to the mixture to stop the reaction. The volume was then filtered through Whatman No. 1 filter paper. The nitrogen content in the filtrate was determined using the micro‐Kjeldahl method, and the digestibility was calculated as:

$$\text{Protein digestibility}\left(\%\right)=\frac{\text{Nitrogen in filtrate}}{\text{Nitrogen in the sample}}\times 100$$

The procedure of Mouliswar, Kurien, Daniel, Malleshi, and Venkatarao (1993) was used to determine the IVSD of the samples. In this assay, 2% of the sample slurry was heated in a water bath at 100°C for 15 min, followed by the addition of 30 ml of 0.2 M glycine‐HCl buffer (pH, 2.0) containing 10 mg pepsin to 50 ml of the slurry. The mixture was incubated at 37°C for 2 h followed by the adjustment of the pH to 7 with 0.2 N NaOH. The final volume was adjusted to 100 ml with distilled water. Five milliliters of 0.5 M phosphate buffer (pH 8.0) containing 15 mg of pancreatin (Carolina) and 15 mg amyloglucosidase (specific gravity: >1, Carolina) was added to 10 ml of the mixture, which was incubated for 2 h at 37°C. After that, the reaction was terminated by heating the mixture in a water bath at 100°C. Then, 2 ml of dinitrosalicylic acid reagent was added to 0.5 ml of the mixture to determine the amount of reducing sugar (Miller, 1959). The standard used was glucose, and a conversion factor of 0.9 was used to calculate the starch equivalent.

### 2.10 Statistical analysis

Three fermentation batches were conducted, and all measurements were done in triplicate. The experiments were designed using a completely randomized block design, and the effects of the treatments on the parameters determined were statistically analyzed using SAS/STAT software (SAS Institute, Cary, NC, USA). Duncan multiple range test with a probability *p *≤* *0.05 was used to separate the means.

## 3 RESULTS AND DISCUSSION

### 3.1 Effect of FBFPF starter levels on chemical composition of raw and fermented Tabat and Wad‐Ahmed sorghum flour

The moisture contents of raw TSF and WSF were 7.73 and 7.78%, respectively (Table 1), which were lower than the range of 8.83–8.76 obtained by Afify, El‐Beltagi, Abd El‐Salam, and Omran (2012) for sorghum flour. Fermentation decreased the moisture contents of both genotypes (*p *≤* *0.05). Incorporation of FBFPF starter at different levels (25%, 50%, 75%, and 100%) in the preparation of fermented sorghum flours had no significant effect on the moisture contents of the flour.

**Table 1:** Chemical composition of raw and fermented Tabat and Wad‐Ahmed sorghum flour prepared with different levels of fermented baobab fruit pulp flour (FBFPF) as starter

Sample	FBFPF (%)	Moisture (%)	Ash (%)	Protein (%)	Fat (%)	Fiber (%)	Carbohydrate (%)
FTSF	0	6.58^b^	1.29^e^	9.88^e^	2.87^b^	3.05^d^	76.33^a^
	25	6.52^b^	1.35^d^	9.96^d^	2.81^b^	3.35^c^	76.01^b^
	50	6.54^b^	1.44^c^	10.00^c^	2.72^c^	3.55^b^	75.75^c^
	75	6.53^b^	1.53^b^	10.26^b^	2.59^d^	3.60^ab^	75.49^d^
	100	6.58^b^	1.59^a^	10.50^a^	2.40^e^	3.70^a^	75.23^e^
Raw TSF		7.73^a^	1.32^de^	9.62^f^	3.03^a^	2.65^e^	75.65^c^
CV%		0.63	1.44	0.17	2.00	2.13	0.16
SE±		0.0350	0.0167	0.0141	0.0447	0.0577	0.0963
LSD_0.05_		0.0762	0.0363	0.0308	0.0873	0.1258	0.1899
FWSF	0	6.48^b^	1.12^e^	9.97^e^	3.17^a^	2.75^c^	76.51^a^
	25	6.40^bc^	1.22^d^	10.26^d^	3.05^b^	2.85^c^	76.22^b^
	50	6.40^bc^	1.30^c^	10.29^c^	2.75^c^	3.10^b^	76.16^b^
	75	6.39^c^	1.39^b^	10.36^b^	2.71^c^	3.20^ab^	75.95^c^
	100	6.43^bc^	1.44^a^	10.56^a^	1.79^d^	3.30^a^	76.48^a^
Raw WSF		7.78^a^	1.21^d^	9.63^f^	3.20^a^	2.35^d^	75.83^d^
CV%		0.63	1.94	0.20	1.68	2.70	0.12
SE±		0.0344	0.0203	0.0168	0.0382	0.0645	0.0742
LSD_0.05_		0.8749	0.0442	0.2366	0.0832	0.1406	0.1116

Mean value(s) having same superscript(s) in a column are not significantly different (*p* ≤ 0.05).

FTSF: fermented Tabat sorghum flour; FWSF: fermented Wad‐Ahmed sorghum flour; TSF: Tabat sorghum flour; WSF: Wad‐Ahmed sorghum flour.

Fermentation significantly lowered (*p *≤* *0.05) the fat and ash contents of raw TSF (3.03% and 1.32% to 2.87% and 1.29%, respectively) and WSF (3.20% and 1.21% to 3.17% and 1.12%, respectively). The fat and ash contents were low compared to previously reported values (Osman, AbdelRahman, Hamad, & Dirar, 2010). The decrease in fat contents after fermentation agreed with that observed in pigeon pea (Adebowale & Maliki, 2011), which may have been due to an increase in the rate of lipolysis to fatty acids and glycerol during the fermentation process. Also, the low ash content observed in fermented sorghum genotypes was different from the high ash contents following fermentation of pearl millet flour, which was attributed to utilization of ash during microbial growth (Azhari, Adiamo, Awad, & Babiker, 2017). A further decrease in fat contents was observed in fermented TSF (FTSF) and fermented Wad‐Ahmed sorghum flour (FWSF) prepared with FBFPF starter. The decrease became significant (*p *≤* *0.05) with increasing FBFPF levels, which could be due to the low‐fat content of baobab fruit pulp flour as reported by Shukla, Dubey, Jain, and Kumar (2001). However, the addition of FBFPF starter to fermented TSF and WSF significantly increased (*p *≤* *0.05) their ash contents over that of raw TSF and WSF, with the highest values obtained in samples treated with 100% FBFPF.

The protein and fiber contents of raw TSF were significantly (*p *≤* *0.05) enhanced after fermentation (9.62% and 2.65% to 9.88% and 3.05%, respectively), as was WSF (9.63% and 2.35% to 9.97% and 2.75%, respectively). These values were lower than the values reported for sorghum flours (Afify et al., 2012; Osman et al., 2010). This increment in the amount of protein during fermentation could be due to solubilization of insoluble proteins of the flours and also the synthesis of protein by microorganisms from metabolic intermediates. The decrease in crude fiber content during fermentation was previously attributed to degradation by fermenting microbes (Babalola & Giwa, 2012), which is contrary to the present findings. Preparation of the fermented sorghum genotypes with FBFPF starter at different levels further significantly increased (*p *≤* *0.05) the protein and fiber contents with increasing FBFPF levels. Abdalla, Hussain, and Mudawi (2010) described that baobab fruit pulp has a protein content range of 5.15%–5.75%, and this could have contributed to the improvement in the protein content of the fermented sorghum flours.

### 3.2 Effect of FBFPF starter levels on mineral contents of raw and fermented Tabat and Wad‐Ahmed sorghum flour

The contents of major minerals and Fe were higher in raw WSF than in TSF genotypes (Table 2). The difference could be due to genetic as well as environmental factors. Fermentation significantly increased the mineral contents for both genotypes, especially P and Mg. The incremental increase in P content during fermentation may have been due to the activity of the phytase microbial enzyme, which hydrolyzes phytate, thereby releasing more P from phenolic compounds. Preparation of FTSF and FWSF with FBFPF starter at different levels significantly enhanced (*p *≤* *0.05) the mineral contents, with FTSF and FWSF having a maximum value of P after treatment with 100% FBFPF. This could be attributed to the high mineral content of baobab fruit pulp. However, incorporation of different levels of FBFPF had no significant effect on the Mg content of both genotypes. Similarly, the Na and P contents of FWSF were significantly (*p *≤* *0.05) improved after addition of FBFPF at levels above 25%. The contents of other minerals were significantly (*p *≤* *0.05) increased even at the lowest levels of FBFPF. The increases were especially pronounced with increased FBFPF levels, with the exception of Fe, where no significant improvement was observed as the levels of FBFPF increased. These values were high as compared to the values obtained with fermented sorghum genotypes flour (Afify et al., 2012).

**Table 2:** Mineral content (mg/100 g) raw and fermented Tabat and Wad‐Ahmed sorghum flour prepared with different levels of fermented baobab fruit pulp flour (FBFPF) as starter

Sample	FBFPF (%)	Na	K	Ca	Mg	P	Fe
FTSF	0	14.50^d^	295.33^e^	24.50^e^	329.50^ab^	325.00^d^	5.95^d^
	25	14.65^c^	305.00^d^	30.73^d^	329.57^a^	330.20 ^cd^	5.96 ^cd^
	50	14.96^b^	314.56^c^	36.35^c^	329.40^b^	336.58^bc^	5.97^bc^
	75	14.93^b^	325.38^b^	40.97^b^	329.48^ab^	341.63^b^	5.98^ab^
	100	15.23^a^	335.47^a^	46.90^a^	329.45^ab^	352.40^a^	5.99^a^
Raw TSF		14.00^e^	275.00^f^	22.50^f^	286.00^c^	295.00^e^	5.91^e^
CV%		0.50	1.59	1.09	0.03	1.37	0.15
SE±		0.0602	4.0053	0.2998	0.0702	3.7039	7.454
LSD_0.05_		0.1313	8.7268	0.6531	0.1529	8.0701	0.0162
FWSF	0	17.62^c^	306.66^d^	28.00^e^	337.72^b^	345.00^c^	5.99^b^
	25	17.71^c^	323.78^c^	33.62^d^	337.45^c^	355.05^bc^	6.01^a^
	50	17.80^b^	335.90^b^	39.25^c^	337.32^c^	353.66^bc^	6.02^a^
	75	17.91^a^	348.35^a^	44.87^b^	336.96^d^	365.16^ab^	6.02^a^
	100	17.97^a^	357.80^a^	50.50^a^	338.00^a^	376.98^a^	6.02^a^
Raw WSF		16.83^d^	276.66^e^	24.00^f^	305.30^e^	315.00^d^	5.95^c^
CV%		1.20	1.82	1.20	0.02	2.02	0.16
SE±		0.1728	4.8181	0.3604	0.0604	5.7925	7.935
LSD_0.05_		0.0864	10.498	0.7852	0.1317	12.621	0.0173

Mean value(s) having same superscript(s) in a column are not significantly different (*p* ≤ 0.05).

FTSF: fermented Tabat sorghum flour; FWSF: fermented Wad‐Ahmed sorghum flour; TSF: Tabat sorghum flour; WSF: Wad‐Ahmed sorghum flour.

### 3.3 Effect of FBFPF starter levels on mineral extractability of raw and fermented Tabat and Wad‐Ahmed sorghum flour

Among the major minerals, the HCl extractability analysis revealed that Na was the most available mineral, while Mg was the least available mineral for both the raw TSF and WSF genotypes (Table 3). HCl extractability of Fe of both raw TSF and WSF was 47.72% and 41.02%, respectively. The HCl extractability of the minerals was enhanced after fermentation of the sorghum genotypes. In a similar study, Sokrab, Mohamed Ahmed, and Babiker (2014) reported that fermentation of high and low phytate corn genotypes improved the HCl extractability of Na and Ca. The improvement in the HCl extractability of the minerals of the two genotypes may be due to the hydrolysis of phytate and tannin by the released phytase and tannase enzymes, respectively. The dephosphorylation mechanism involves the removal of phosphate groups from the inositol ring of phytate, which decreases the strength of mineral binding to phytate, thereby enhancing the bioavailability of essential minerals (Sandberg et al., 1999). The use of FBFPF starter in the preparation of the fermented samples further significantly increased the HCl extractability of the minerals (*p *≤* *0.05). The mineral extractability of both genotypes increased progressively and significantly (*p *≤* *0.05) with the starter (FBFPF) levels, except for Na, K, and Ca, which were extracted best in samples prepared with 75% FBFPF. This increase could be attributed to the high mineral extractability of the baobab fruit pulp flour (Abdalla, Mohammed, et al., 2010). For FTSF, the HCl extractability of Mg increased from 51.22% to 61.95%, while that of FWSF increased from 48.82% to 53.59% after preparation of the sample with 100% FBFPF. Furthermore, the iron extractability of FTSF and FWSF prepared with 100% FBFPF was also enhanced (*p *≤* *0.05) to 51.44% and 41.02%, respectively. The obtained values were high as compared to that of fermented sorghum genotypes flour reported by Idris et al. (2005).

**Table 3:** Mineral extractability (%) of raw and fermented Tabat and Wad‐Ahmed sorghum flour prepared with different levels of fermented baobab fruit pulp flour (FBFPF) as starter

Sample	FBFPF (%)	Na	K	Ca	Mg	P	Fe
FTSF	0	83.10^e^	78.93^b^	48.00^e^	51.22^e^	60.60^e^	49.49^e^
	25	83.29^d^	79.89^a^	48.53^d^	52.27^d^	62.19^c^	50.00^d^
	50	83.50^c^	78.49^c^	49.03^c^	56.52^c^	62.61^a^	50.40^c^
	75	83.69^b^	76.70^e^	50.62^b^	59.18^b^	62.41^b^	51.38^b^
	100	83.77^a^	75.25^f^	50.86^a^	60.78^a^	61.95^d^	51.44^a^
Raw TSF		78.56^f^	77.59^d^	46.64^f^	39.62^f^	41.40^f^	47.72^f^
CV%		0.03	0.02	0.08	0.47	0.05	0.16
SE±		0.0208	0.0120	0.0317	0.2027	0.0216	0.0643
LSD_0.05_		0.0454	0.0262	0.0690	0.4417	0.0471	0.1401
FWSF	0	92.52^b^	73.53^e^	50.25^e^	48.82^e^	52.77^d^	49.50^e^
	25	92.55^b^	76.90^a^	55.73^a^	49.94^d^	55.45^c^	50.83^d^
	50	92.32^c^	75.62^b^	54.06^c^	51.20^c^	55.62^b^	50.90^c^
	75	92.65^a^	75.51^c^	54.53^b^	52.36^b^	55.45^c^	51.21^b^
	100	92.34^c^	74.22^d^	53.98^d^	53.59^a^	56.55^a^	51.60^a^
Raw WSF		86.53^d^	65.51^f^	41.67^f^	38.09^f^	38.71^e^	41.02^f^
CV%		0.02	0.04	0.04	0.05	0.05	0.06
SE±		0.0186	0.0243	0.0186	0.0186	0.0219	0.0236
LSD_0.05_		0.0404	0.0530	0.0404	0.0404	0.0476	0.0514

Mean value(s) having same superscript(s) in a column are not significantly different (*p* ≤ 0.05).

FTSF: fermented Tabat sorghum flour; FWSF: fermented Wad‐Ahmed sorghum flour; TSF: Tabat sorghum flour; WSF: Wad‐Ahmed sorghum flour.

### 3.4 Effect of FBFPF starter levels on pH, TA, and microbiological characteristics of raw and fermented Tabat and Wad‐Ahmed sorghum flour

**Figure 1:**
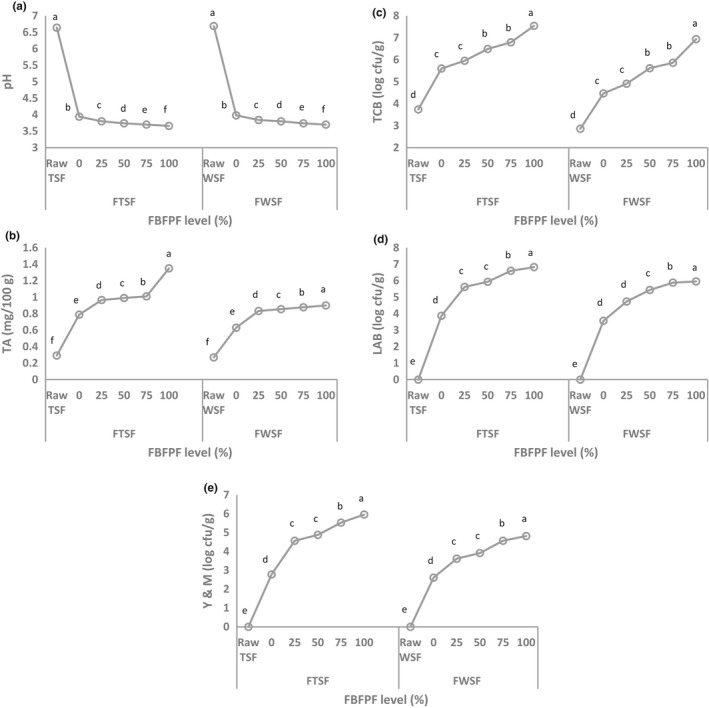
pH, TA and microbiological properties of raw and fermented Tabat and Wad‐Ahmed sorghum flour prepared with different levels of fermented baobab fruit pulp flour (FBFPF) as starter. LAB: lactic acid bacteria; TA: total acidity; TCB: total count of bacteria; YM: yeast and mold

The initial pH of Tabat (6.64) and Wad‐Ahmed (6.69) flours was significantly lowered (*p *≤* *0.05) after fermentation without the addition of FBFPF starter to 3.94 and 3.98, respectively (Figure 1a). These could be due to the formation of acids during fermentation of the samples. These results agreed with those obtained by Alka, Neelam, and Shruti (2012) who reported that fermentation causes a rapid drop in pH of sorghum, pearl millet, and maize. The pH values were higher than the range of 5.8–6.0 obtained by AbdelRahman, Hamad, Osman, and Dirar (2010) for sorghum flours. These differences can be attributed to environmental factors, such as soil, climate, maturity, and season of collection of the sorghum grains. Figure 1a also indicates that the incorporation of FBFPF starter resulted in a further decline in the pH values, which decreased as FBFPF levels increased. Baobab pulp fruit reportedly has a low pH (3.03–3.20; Abdalla, Mohammed, et al., 2010), which might lead to a reduction in the pH of the FTSF and FWSF when used as starter.

Fermentation raised the TA of raw TSF from 0.293 to 0.788 mg/100 g and of WSF from 0.27 to 0.73 mg/100 g (Figure 1b). These values were higher than that of sorghum flour (0.22 mg/100 g) reported by AbdelRahman et al. (2010). Further increase in TA was observed in fermented samples treated with FBFPF starter, with the increase becoming significant (*p *≤* *0.05) with increasing levels of FBFPF. A similar observation was reported by Nnam and Obiakor (2003). The decrease in pH with a concomitant increase in acidity may be due to the activity of LAB.

Microbiological characterization (Figure 1c–e) revealed that raw TSF and WSF had the least microbial counts (LAB, TCB, and YM) compared to fermented samples. A sharp increase in YM counts was observed following the addition of 25% FBFPF to FTSF and FWSF. However, the addition of the same level of FBFPF to FTSF and FWSF had no significant effect on the TCB and LAB of the samples. Increasing the levels of FBFPF starter significantly (*p *≤* *0.05) increased the microbial loads, particularly LAB, in the fermented samples. The observation that baobab fruit pulp fermentation was mainly lactic acid fermentation agreed with Parkouda et al. (2010) who found that *Enterococcus faecium* was the predominant microorganism during fermentation of baobab seeds.

### 3.5 Effect of FBFPF starter levels on antinutritional factors, IVPD, and IVSD of raw and fermented Tabat and Wad‐Ahmed sorghum flour

**Figure 2:**
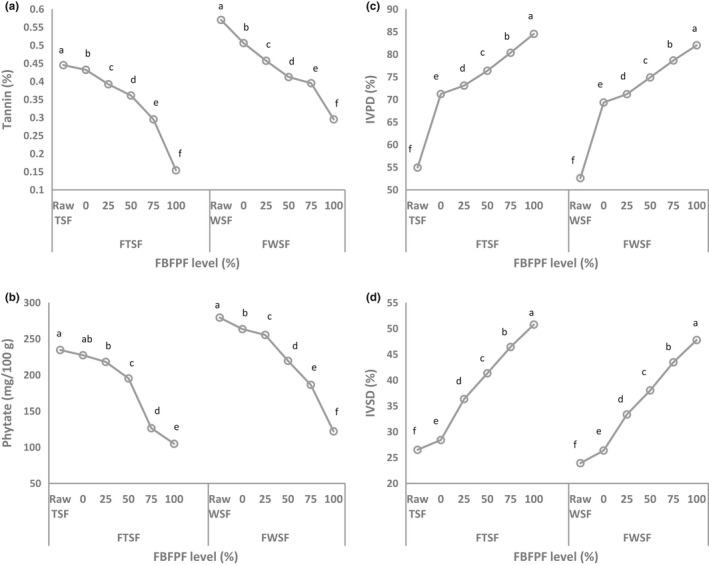
Antinutrients and in vitro protein and starch digestibility of raw and fermented Tabat and Wad‐Ahmed sorghum flour prepared with different levels of fermented baobab fruit pulp flour (FBFPF) as starter. IVPD: in vitro protein digestibility; IVSD: in vitro starch digestibility

The effects of FBFPF levels on the antinutritional factors, IVPD, and IVSD of raw and fermented TSF and WSF are presented in Figure 2. Raw TSF and WSF displayed the highest contents of tannin (0.445% and 0.57%, respectively) and phytate (234.57 and 279.33 mg/100 g, respectively) (Figure 2a,b). The IVPD of raw TSF and WSF was 54.93% and 26.50%, respectively (Figure 2c). The IVSD of raw TSF and WSF was 52.59% and 23.92%, respectively (Figure 2d). The values of IVPD were within the range of 49.25%–55.85% reported by Awadelkareem, Muralikrishna, El Tinay, and Mustafa (2009) for sorghum flours.

Figure 2 revealed that fermentation significantly (*p *≤* *0.05) reduced the antinutritional factors contents, except for the phytate content of FTSF. Also, further significant reduction (*p *≤* *0.05) occurred after addition of FBFPF, with the decrease becoming more pronounced with increased levels of FBFPF starter. However, significant increases in IVPD and IVSD were observed in the fermented samples (*p *≤* *0.05). Moreover, sharp rises in IVSD and IVPD were evident following the incorporation of FBFPF, with further increases occurring as the FBFPF level increased. The decreased level of phytic acid of fermented sorghum grains has been reported (Idris et al., 2005) due to the action of the phytase enzyme released during fermentation. Also, the reduction in the level of tannin in fermented samples may be attributed to the action of tannase produced by microorganisms during fermentation (Barthamuef, Regerat, Pourrat, & Pourrat, 1994). The increase in IVPD values with the length of fermentation agrees with the findings of Mohiedeen, Tinay, Elkhalya, Babiker, and Mallasiy (2010) for fermented maize cultivars. The authors attributed the increase to the partial degradation of complex storage proteins to simpler soluble products. According to Hassan and El Tinay (1995), fermentation can cause changes in the fractions of endosperm protein, which renders starch more accessible to the digestive enzyme, which may result in an increased IVSD.

### 3.6 Effect of FBFPF starter levels on functional and pasting properties of raw and fermented Tabat and Wad‐Ahmed sorghum flour

**Figure 3:**
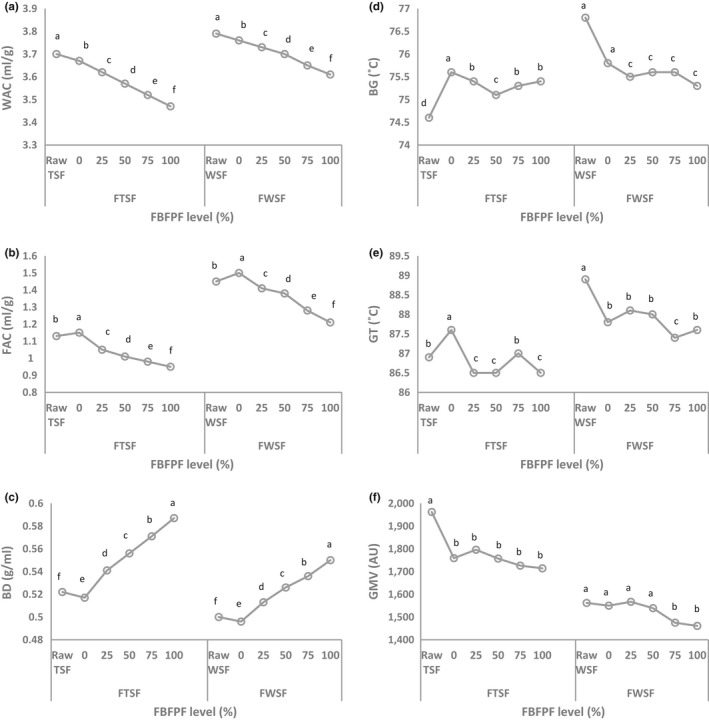
Functional (a–c) and pasting properties (d–f) of raw and fermented Tabat and Wad‐Ahmed sorghum flour prepared with different levels of fermented baobab fruit pulp flour (FBFPF) starter. BG: beginning of gelatinization; FAC: fat absorption capacity; GT: gelatinization temperature; GMV: gelatinization maximum viscosity; WAC: water absorption capacity

Figure 3a–c summarizes the effect of FBFPF addition at different levels on the functional properties of raw and fermented TSF and WSF. Fermentation significantly decreased (*p *≤* *0.05) the WAC and BD of raw TSF (3.7 ml/g and 0.522–3.67 ml/g and 0.517, respectively) and WSF (3.79 ml/g and 0.5–3.76 ml/g and 0.496, respectively). The difference in WAC between the two sorghum genotypes may reflect differences in the nature of hydrophilic constituents. Decreased WAC and BD of fermented samples were reported for fermented sorghum flour by Alka et al. (2012). A further significant decrease (*p *≤* *0.05) in WAC was observed following the addition of FBFPF at different levels. This decline was reduced with an increase in FBFPF levels. However, the BD of FTSF and FWSF increased (*p *≤* *0.05) as the level of FBFPF increases. The FAC values of raw and fermented WSF were lower than that of TSF. Also, raw TSF and WSF displayed FAC values lower than their fermented samples without FBFPF addition. This result agrees with that of fermented *Hura crepitan* seeds (Osungbade, Gbadamosi, & Adiamo, 2016). FAC is necessary as it improves mouthfeel due to the retention of flavor by oil and also bestows a soft texture to food (Ubbor & Akobundu, 2009). Incorporation of FBFPF reduced the FAC of the fermented samples with lowest value obtained after the addition of 100% FBFPF. This was probably due to the low‐FAC of FBFPF.

Figure 3d–f summarize the beginning of gelatinization (BG), gelatinization temperature (GT), and gelatinization maximum viscosity (GMV) of raw and fermented TSF and WSF as influenced by the incorporation of different levels of FBFPF. Raw WSF displayed higher BG and GT than raw TSF. Pasting temperature for raw TSF (74.6°C) and WSF (76.8°C) was reduced to 75.1°C and 75.8°C, respectively, after fermentation. The maximum pasting temperature was evident in fermented samples prepared without the addition of FBFPF starter, while the lowest pasting temperature was evident in FTSF and FWSF treated with 50% and 100% FBFPF starter. The maximum GTs for raw TSF and WSF were 86.9°C and 88.9°C, respectively. Fermentation decreased the GT of raw WSF and increased that of raw TSF without FBFPF starter (both *p *≤* *0.05). Incorporation of FBFPF starter at different levels varied the GT of the samples with lowest values obtained in those treated with 100% FBFPF starter. Unlike BG and GT, the GMV of TSF was higher than that of WSF. A sharp decline in GMV was noted after fermentation of raw TSF. Similar to GT, the GMV value varied after treatment with FBFPF starter at different levels, with the lowest values were obtained in fermented samples containing 100% FBFPF starter.

## 4 CONCLUSION

The FBFPF as a starter in the fermentation of sorghum flour substantially lowers the antinutritional factors of the fermented product. Furthermore, fermentation with FBFPF starter is valuable in improving the nutritional, total and extractable minerals, and functional properties of sorghum flour. Further research should specifically address the proportional incorporation of FBFPF starter in cereal‐based traditional foods, such as *Assida*,* Kisra*, and *Injera* to improve the nutritional qualities of these staple foods.

## CONFLICT OF INTEREST

Authors declare that there is no conflict of interests.

## ETHICAL STATEMENT

This work does not involve any human or animal experiments.
